# Recessive genetic mode of an *ADH4 *variant in substance dependence in African-Americans: A model of utility of the HWD test

**DOI:** 10.1186/1744-9081-4-42

**Published:** 2008-09-18

**Authors:** Xingguang Luo, Lingjun Zuo, Henry R Kranzler, Shuang Wang, Raymond F Anton, Joel Gelernter

**Affiliations:** 1 Department of Psychiatry, Yale University School of Medicine, New Haven, CT, USA; 2 VA Connecticut Healthcare System, West Haven Campus, CT, USA; 3 Alcohol Research Center, Department of Psychiatry, University of Connecticut School of Medicine, Farmington, CT, USA; 4 Department of Biostatistics, Mailman School of Public Health, Columbia University, New York, NY, USA; 5 Alcohol Research Center, Institute of Psychiatry, Medical University of South Carolina, Charleston, SC, USA

## Abstract

**Background:**

In our previous studies, we reported positive associations between seven *ADH4 *polymorphisms and substance dependence [i.e., alcohol dependence (AD) and/or drug dependence (DD)] in European-Americans (EAs). In the present study, we address the relationship between *ADH4 *variation and substance dependence in an African-American (AA) population, and report evidence that supports an association between a different *ADH4 *polymorphism (rs2226896) and these phenotypes in AAs.

**Methods:**

Two family-based association study methods, i.e., TDT and FBAT, were applied to test the relationship between *ADH4 *variation and substance dependence in Sample 3 (112 small nuclear families) and in Sample 4 (632 pedigrees), respectively. A population-based case-control association study method was also applied to test this relationship in 1303 unrelated subjects, with and without controlling for admixture effects. Finally, a Hardy-Weinberg Disequilibrium (HWD) test was applied to examine the association in the case-only sample, infer the genetic disease models, and distinguish the disease and non-disease factors contributing to HWD.

**Results:**

The marker examined was found to be in significant HWD in AA alcoholics (p = 0.0071) and drug dependent subjects (p = 0.0341), but in Hardy-Weinberg Equilibrium (HWE) in all other subgroups. Other association methods failed to detect any association between this variation and phenotypes. The best-fit genetic disease model for this marker is a recessive genetic model.

**Conclusion:**

*ADH4 *variation might play a role in risk for substance dependence in AAs, potentially via a recessive mechanism. Under certain conditions, the HWD test could be a more powerful association method than conventional family-based and population-based case-control association analyses, for which, the present study provides an extreme example.

## Background

The rate of alcohol metabolism influences drinking behavior and affects risk for alcohol dependence (AD). The alcohol dehydrogenases (ADH) and the aldehyde dehydrogenases (ALDH) are the two major categories of alcohol-metabolizing enzymes in the liver. ADH converts alcohol to acetaldehyde (which is toxic) and then ALDH converts acetaldehyde into acetate. Acetate is then oxidized into CO_2 _and H_2_O via the tricarboxylic acid cycle. The development of AD is related to an individual's level of ethanol consumption, which is influenced by ADH and ALDH activity. The human ADH4 enzyme (i.e., π subunit) is an important member among these alcohol-metabolizing enzymes. It mainly contributes to liver ADH activity, and at intoxicating levels of alcohol it may account for as much as 40% of the total ethanol oxidation rate [1]. Thus, physiologically significant variation in ADH4 activity could clearly contribute to variability in risk for AD.

The human ADH4 enzyme is encoded by the *ADH4 *gene, which maps to 4q22 within the ADH gene cluster. We focused on the *ADH4 *gene in this study because of the functional importance of its protein product, remarkable for its activity at high ethanol concentrations that might be of particular relevance in the context of AD risk [2]; and because previous studies have demonstrated that *ADH4 *is an important risk gene for AD and AD-related traits in European-Americans (EAs) and African-Americans (AAs). Guindalini et al. [3] reported that the *ADH4 *promoter variants -75A/C (rs1800759), which can significantly alter the expression of the ADH4 enzyme [4], and -159A/G, were significantly associated with AD in EAs and AAs in a Brazilian population. Edenberg et al. [5] reported that sixteen *ADH4 *polymorphisms (including rs2226896) were associated with AD in an independent Collaborative Study on the Genetics of Alcoholism (COGA) sample of pedigrees. We have previously reported associations between seven *ADH4 *polymorphisms (including -75A/C) and AD and drug dependence (DD) in EAs in the US population [6,7]. We also reported that three *ADH4 *polymorphisms (including -75A/C) were associated with personality traits in AD and DD subjects [8].

In our previous studies [6,7], we genotyped seven markers in some of the subjects included in the present study. These seven markers span the full length of *ADH4 *and are in one haplotype block both in EAs and AAs. The seven *ADH4 *markers showed deviation from Hardy-Weinberg Equilibrium (HWE) (called Hardy-Weinberg Disequilibrium, HWD) in EA substance dependent subjects (including patients with AD and DD), but were in HWE in EA healthy controls. Significant differences in genotype and diplotype, but not in any allele or haplotype, frequency distributions for all seven *ADH4 *markers were found between cases and controls in EAs (adjusted global p = 0.0070, 0.0004 for AD and DD, respectively). We also demonstrated that *ADH4 *genotypes predispose to AD and DD consistent with a recessive mode of inheritance in EAs. These associations remained after controlling for admixture effects and were confirmed by a family-based association study. However, in AAs, these seven *ADH4 *markers were in HWE in both cases and controls; no association between alleles, genotypes, haplotypes, or diplotypes of these markers and AD and DD were found in this population, even after controlling for admixture effects.

Although we did not find a significant association between these seven *ADH4 *variants and AD or DD in AAs in our previous studies, in view of the power variance of association methods and the population-specificity of associations, we did not exclude the possibility that these associations might be significant if adequate statistical power were available, or that other markers might be associated with these phenotypes in this population [6,7]. The present study aimed to identify an association between a specific polymorphism, rs2226896 (for convenience and to distinguish it from the seven polymorphisms on which we reported previously, we refer to this variant as SNP8) and AD, in AAs. This polymorphism has a rare allele and a rare homozygote which could result in its being independent of the flanking haplotype block and could increase the relative risk between genotypes so that it could be a more powerful marker for some specific association analysis methods (e.g., the HWD test) to detect marker-phenotype association [9]. To test the population-specificity of this association, we also tested for it in EAs, the most common population in the US.

As different association methods have different advantages, disadvantages, and power associated with them, in the present study, we applied several methods and compared their power. Family-based association study methods are immune to population stratification and admixture effects, and (as generally applied) are not informed by the affection status of parents. This method has been used as a valid confirmatory method for a population-based association study in our previous study [6] and was applied in the present study as well. However, because the sample size of families is usually limited, and families usually provide around two-thirds of the power provided by unrelated case-control samples of similar size [10], we also performed a case-control association study. AAs are an admixed population, and EAs are admixed as well, although much less so [11-14]. Admixture effects may result in spurious observed association between gene and disease. A population-based case-control association study is vulnerable to these effects. Therefore, the degree of admixture was also measured in the case-control sample, and admixture effects on the case-control association analysis were controlled by a structured association (SA) method [15].

Our previous study suggested that a case-only HWD test was more powerful (with lower p values) than a case-control association analysis under a recessive model [7]. The present study provided an additional application for this powerful association method. Meanwhile, we used an HWD test to infer the genetic disease model, via a set of novel software programs developed by Wittke-Thompson et al. [9], which incorporate the HWD information from both cases and controls. HWE is subject to a number of assumptions regarding genotyping error, selection, genetic drift, inbreeding, gene flow, and mutation, and to other factors such as population stratification, admixture, nonrandom patterns of missing data, and nonrandom allele dropout with increasing age. HWD may be caused by the violation of any one of these assumptions; thus, we also distinguished the underlying causes for HWD by the above methodology via application of the recently-developed programs and by reviewing almost all possibility for the causes of HWD in details.

Drug dependence (DD), which mainly includes cocaine dependence and opioid dependence in our sample, is one of the most common phenotypes comorbid with AD [16]. DD has many features in common with AD, including symptomatology, neuropsychological impairment, hypothesized pathogenetic mechanisms, and response to specific treatments (reviewed by Luo *et al *[7]). DD has also been reported to share some susceptibility genes with AD (reviewed by Luo *et al *[6,7,17]). Our previous study demonstrated that DD and AD share *ADH4 *as a susceptibility gene in EAs [6,7]. Thus, in this study, we investigated the association not only in AD, but also in DD, to test the phenotypic specificity of the observed associations.

## Methods

### 1. Subjects

The clinical samples are listed in Tables 1 and 2, including Sample 1 (907 unrelated case-control subjects), Sample 3 (112 small nuclear families (SNFs); each family includes parents and 1 to 2 offspring) [both samples were used in previous studies by Luo *et al *[6,7]], Sample 2 (271 newly recruited unrelated AA cases and 125 unrelated AA controls), and Sample 4 (1613 related subjects from 632 pedigrees; each pedigree has 1 to 6 affected siblings with or without parents) [Sample 4 was previously described by Gelernter *et al *[18,19]]. Samples 1 and 2 included a total of 1303 unrelated subjects (757 males and 546 females), with 391 "genetic" EA cases, 310 "genetic" EA healthy controls (European ancestry proportion > 0.5), 429 "genetic" AA cases, and 173 "genetic" AA controls (African ancestry proportion > 0.5; see Methods). Newly-recruited subjects (Sample 2) were diagnosed using the Semi-Structured Assessment for Drug Dependence and Alcoholism (SSADDA) [18,20]. The healthy control subjects (in Samples 1 and 2) were screened to exclude major Axis I mental disorders, including alcohol or drug use disorders, psychotic disorders (including schizophrenia or schizophrenia-like disorders), mood disorders, and major anxiety disorders. Sample 4 included pedigrees having affected probands with substance dependence, previously used for genome-wide linkage studies [18,19]. Diagnosis was made according to DSM-III-R or DSM-IV criteria [21,22].

**Table 1:** Sample size for the unrelated sample and the related small nuclear families.

				Sample sizes			
				
		%Male	Age (years)	EA	AA	Hispanic	Others
Sample 1	Healthy controls	39.9	28.2 ± 9.1	310	48		
	Cases	73.4	39.4 ± 9.2	391	158		
	AD	76.1	40.3 ± 9.2	326	101		
	DD	68.2	37.1 ± 8.1	204	145		
Sample 2	Healthy controls	28.0	33.5 ± 12.6		125		
	Cases	64.9	40.0 ± 9.3		271		
	AD	65.1	40.0 ± 9.3		269		
	DD	64.8	42.0 ± 8.3		71		
Sample 3	SNF size			92	12	6	2
	Parents	49.8	65.9 ± 8.6	183	24	11	3
	%Unaffected	45.5	66.8 ± 8.6	79.8	37.5	63.6	100.0
	Offspring	54.8	39.2 ± 11.1	96	13	5	1
	AD	58.9	37.3 ± 8.2	77	9	3	1
	DD	55.2	38.0 ± 7.3	77	13	5	1

EA, European-American; AA, African-American; AD, alcohol dependence; DD, drug dependence; SNF, small nuclear families. Samples 1 and 2 are unrelated case-control samples; Sample 3 is related SNF sample.

**Table 2:** Structure and sizes of pedigrees (Sample 4)

	Total	EAs	AAs	Others
Subjects	1613	766	833	14
%Male	46.3	51.4	41.2	71.4
Age (year)	39.4 ± 8.9	37.8 ± 10.2	40.8 ± 7.2	43.7 ± 5.7
Pedigrees	632	311	316	5
With 1 AD	242	115	126	1
With 2 AD	166	87	78	1
With 3 AD	11	5	6	0
With 4 AD	5	2	3	0
With ≥ 5 AD	1	0	1	0
With 1 DD	57	25	32	0
With 2 DD	460	243	216	1
With 3 DD	90	35	55	0
With 4 DD	16	7	9	0
With ≥ 5 DD	3	0	3	0

EA, AA, AD, DD, same as Table 1. "With n AD", a pedigree having n offspring with AD; as analogy.

Samples 1, 2 and 3 were recruited at the University of Connecticut Health Center, the VA Connecticut Healthcare System-West Haven Campus, or the Medical University of South Carolina. Sample 4 was recruited at four sites: University of Connecticut Health Center (Farmington, CT), Yale University School of Medicine (New Haven, CT), McLean Hospital, Harvard Medical School (Belmont, MA), and Medical University of South Carolina (Charleston, SC). All subjects gave informed consent before participating in the study, which was approved by the Institutional Review Board at the respective institutions.

### 2. Genotyping

A marker (rs2226896; SNP8; at Chr04: 100460117) at the putative 5' regulatory region, close to the functional variant -75A/C (at Chr04: 100458289; 1.8 Kb to 3' of SNP8) at the promoter and other disease-related polymorphisms at this region (e.g., -159G/A at Chr04: 100458373, 1.7 Kb to 3' of SNP8 [3]; rs1984362 at Chr04: 100463753, 3.6 Kb to 5' of SNP8 [6,7]) was genotyped in all subjects using the TaqMan technique ("assay-on-demand"). These eight ADH4 SNPs were all of those that were available from public sources when we started genotyping. SNP8 was not reported together with other seven SNPs in our previous studies [6,7] because it was not associated with any phenotype in EAs. The detailed genotyping procedure is described elsewhere [7]. All genotyping was performed in duplicate and compared to ensure validity of the data. Mismatched genotypes, if any, were discarded. Thirty-eight unlinked ancestry-informative markers (AIMs) were also genotyped in unrelated subjects to estimate the ancestry proportions for each subject (detailed by Luo *et al *[6]).

### 3. Statistical analysis

1) Linkage disequilibrium (LD) analysis: Pairwise LD between this marker and the other seven *ADH4 *markers studied previously was analyzed separately for EAs and AAs in Sample 1 via the program Haploview [23]. The additional newly recruited 271 AA unrelated cases and 125 unrelated AA controls (Sample 2), and the related pedigree subjects (Sample 4) were not included in this analysis because the other flanking seven *ADH4 *markers have not been genotyped in these subjects.

2) Transmission disequilibrium test (TDT): Family-based association analysis was performed by comparing "cases" (i.e., transmitted allele and genotypes) and artificial "controls" (i.e., untransmitted allele and genotypes) in Sample 3 by the program TDTPHASE [24]. All subjects in Sample 3, including EA, AA, Hispanic, and others, and the affected and unaffected parents, were combined in the TDT analysis. This analysis was also performed separately within EAs and AAs.

3) Family-Based Association Tests (FBAT): The structure of Sample 4 is much more complicated than Sample 3, thus, we used the program FBAT [25] to test the gene-AD and gene-DD associations under the general, additive, dominant, and recessive genetic models respectively. We have already genotyped 419 short tandem repeats (STRs) in this sample in the initial whole genome scan linkage studies [18,19], and inferred the ancestry proportions in each individual. The whole sample can thus be separated into "genetic" EAs (European ancestry proportion > 0.5) and "genetic" AAs (African ancestry proportion > 0.5), according to the ancestry proportions in probands. FBAT was performed in the whole sample including EAs, AAs, Hispanics, and others. This analysis was also performed separately within "genetic" EAs and AAs, because the population-specificity of the gene-disease linkage and the disease-risk sites has ever been demonstrated in this sample [18,19].

4) Case-control comparisons for allele and genotype frequencies: Allele and genotype frequencies for SNP8 among EAs and AAs in Samples 1 and 2 are shown in additional file 1. Associations between alleles, genotypes and phenotypes were tested by comparing the allele and genotype frequency distributions between cases and controls with the exact tests in the program PowerMarker [26].

5) Structured association (SA) analysis: EAs and AAs were taken as admixed populations with different degrees of admixture. The extent of admixture (i.e., average ancestry proportions) in unrelated subjects was estimated using the program STRUCTURE [15] by analyzing 38 AIMs (in combined EA and AA subjects) [6,27,28]. Admixture effects on case-control association analysis can be controlled using the program STRAT [29] by conditioning the analysis on the ancestry proportions, i.e., structured association (SA) analysis (separately for EAs and AAs). Furthermore, associations between this *ADH4 *variation and phenotypes were also analyzed by a regression method (in combined EAs and AAs), with ancestry proportions, age, and sex serving as covariates.

6) Hardy-Weinberg equilibrium (HWE) test and genetic disease model inference: An HWE test can be a valid association method, and can be used to infer genetic disease models. HWE was tested within populations, and separately for cases and controls, using the goodness-of-fit χ^2 ^test implemented in the program PowerMarker [26]. If there was one cell with expected genotype count less than 5, HWE was also tested by the exact test implemented in the program PowerMarker [26] and confirmed by the exact test implemented in the online program FINETTI [30], and the exact tests implemented in the program TFPGA [31] which applies conventional Monte Carlo and Markov Chain Monte Carlo (MCMC) methods.

To distinguish the causes of HWD, which include gene-disease association and non gene-disease association factors such as genetic drift, migration, mutation, non-random mating, etc., and then to infer the genetic disease model for the marker in HWD, we used the goodness-of-fit *χ*^2 ^test defined by Wittke-Thompson et al. [9], which incorporated the HWE information both from cases and controls (here denoted WT goodness-of-fit *χ*^2 ^test) [9]. The test statistic $\chi^{2}=\sum_{i}\frac{{\left(O_{i}-E_{i}\right)}^{2}}{E_{i}}+\sum_{j}\frac{{\left(O_{j}-E_{j}\right)}^{2}}{E_{j}}$ (*i*, *j *= 1, 2 or 3), where *O*_*i *_and *E*_*i *_are the observed and expected numbers of *i*^*th *^genotype of this marker in patients, respectively; *O*_*j *_and *E*_*j *_are the observed and expected numbers of *j*^*th *^genotype of this marker in controls, respectively. Assuming different baseline penetrance of disease (from 0 to 1) for non-susceptibility homozygotes, different heterozygote relative risk, and different susceptibility homozygote relative risk that determine different *E*_*i *_and *E*_*j *_given different genetic models, and setting the lifetime population prevalence of alcohol dependence and drug dependence at 5.5% and 3.8% [32], respectively, we can obtain a different set of *χ*^2 ^values. Minimizing this *χ*^2 ^statistic over the entire parameter space with the appropriate constraints on the parameters approximates a maximum-likelihood estimate procedure. We can then obtain the minimal (*χ*^2^)_0 _value, which is approximately distributed as a *χ*^2 ^with 1 *df *for a general model and 2 *df *for restricted models (i.e., dominant, recessive, additive, and multiplicative models) [33]. Simulating 173 AA controls and 370 AA AD subjects or 216 AA DD subjects for 1000 replications to obtain 1000 minimal *χ*^2 ^values and comparing these values to the original minimal (*χ*^2^)_0 _value, we can derive an empiric p-value. If this p-value is less than 0.05, the genetic disease model can be rejected as a poor fit to the observed data. The best-fit genetic model would correspond to a p-value which is larger than 0.05 and is the largest among all genetic models. If all of the genetic models are poorly fitted, then alternative explanations for the HWD, including chance, genotyping error, and/or violations of the requisite assumptions of HWE, must be considered.

The direction of HWD statistics (Δ, F and J values, detailed definitions follow) in cases is also helpful to judge the genetic models. Under the dominant and the additive models, the values of Δ, F and J are negative, because HWD is caused by an excess of observed heterozygote (Aa) and homozygote (AA) frequencies over the expected frequencies. Under the recessive model, the values of Δ, F and J are positive, because the HWD is caused by excess of observed homozygote (aa) frequency over the expected frequency, and in contrast, observed heterozygote (Aa) frequency is less than expected. These statistics were calculated based on the following formula: Assuming that *fo*', *fo*", *fo*"', and *fe*', *fe*", *fe*"' denote the **o**bserved and **e**xpected frequencies of susceptibility homozygote, heterozygote and non-susceptibility homozygote in cases, respectively, then Δ = *fo*'''-*fe*''', F = 2Δ/*fe*'' and J = Δ/*fe*"' [34-36].

## Results

**Figure 1:**
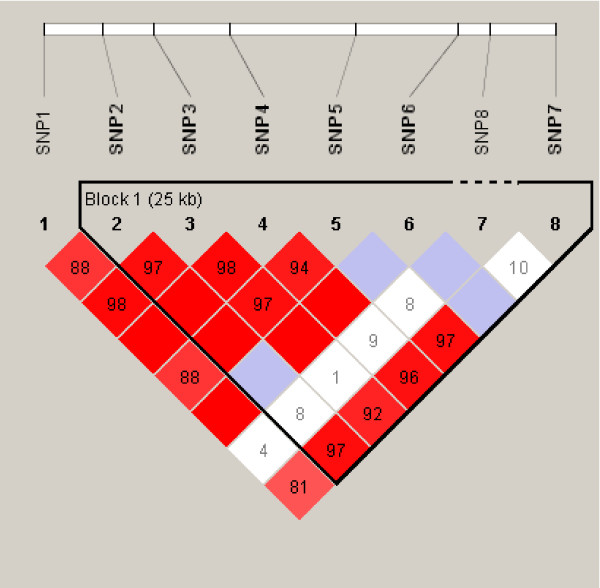
**Pairwise LD between the candidate marker SNP8 and other seven flanking markers in unrelated African-American subjects**. [D' = 1.00 in the blank squares; the numbers inside the red squares are D' × 100%; the blue squares represent low r^2 ^values; the white squares represent low D' and low r^2 ^values. SNPs 1–7 were reported previously in Luo et al. (2006) and span from 3' to 5'.].

1. SNP8 was in LD with the seven flanking markers in EAs (D' > 0.9 in each case), but provided information independent of these markers in AAs (D' < 0.10 in each case) (Figure 1). TDT in Sample 3 and FBAT in Sample 4 showed no significant association between the *ADH4 *SNP8 and AD or DD, whether analysis is conducted by combining or separating the different ethnicities (all p > 0.05) (Sample 3: see additional file 2; Sample 4: data not shown). The genotype frequency distributions in Sample 4 were shown in Table 3, not for the association analysis, but for providing a replication of the rare genotype frequencies to those in the unrelated Samples 1 and 2 (see additional file 1).

**Table 3:** Genotype frequency distributions of SNP8 in pedigrees (Sample 4)

	European-American						African-American					
		
	AD		DD		Unaffected		AD		DD		Unaffected	
						
	*N*	*f*	*N*	*f*	*N*	*f*	*N*	*f*	*N*	*f*	*N*	*f*
A/A	271	0.903	541	0.905	85	0.895	293	0.945	626	0.963	127	0.992
A/G	26	0.087	53	0.089	8	0.084	16	0.052	24	0.037	1	0.008
G/G	3	0.010	4	0.007	2	0.021	1	0.003	0	0.000	0	0.000
A	568	0.947	1135	0.949	178	0.937	602	0.971	1276	0.982	255	0.996
G	32	0.053	61	0.051	12	0.063	18	0.029	24	0.018	1	0.004

AD, alcohol dependence; DD, drug dependence.

2. SNP8 was not associated with phenotypes via case-control comparisons either before or after controlling for admixture effects. Measured admixture degrees were low both in EAs and AAs.

No significant difference in allele or genotype frequency distribution for this marker was found between unrelated cases and controls in either EAs or AAs (all p > 0.05; additional file 1). After controlling for admixture effects by the SA method, these negative associations were essentially unchanged (all p > 0.05). Regression analysis, which takes into account the effects of age and sex, also showed no significant association.

Two main ancestries, i.e., European and African, were detected in our sample. In the 701 "genetic" EA subjects (European ancestry proportion > 0.5), the total estimated weight of African ancestry proportions is 11.3; the admixture degree of "genetic" EAs in this sample is 1.6% (= 11.3/701). In the 602 "genetic" AA subjects (African ancestry proportion > 0.5), the total estimated weight of European ancestry proportions is 23.7; the admixture degree of "genetic" AAs is 3.9% (= 23.7/602).

3. SNP8 was in significant HWD in both AA AD and DD subjects (with an excess of observed homozygotes over expected homozygotes): using the exact tests: p = 0.0071 for AD and p = 0.0341 for DD, respectively. These HWDs remained significant or suggestive after correction for multiple testing (α was set at 0.025). However, SNP8 was in HWE in EA AD and DD subjects (p > 0.05) (additional file 1). This marker was in HWE in controls, both EA and AA (p > 0.10). The WT goodness-of-fit *χ*^2 ^test showed that the best-fit genetic disease model for this marker was a recessive model (in AA AD subjects: *χ*^2 ^= 0.621, *df *= 2, p = 0.733; in AA DD subjects: *χ*^2 ^= 1.596, *df *= 2, p = 0.450; see additional file 1). In AA AD subjects for this marker, Δ = +0.008, F = +0.182 and J = +0.009; in AAs with drug dependence, Δ = +0.008, F = +0.204 and J = +0.010.

## Discussion

In African-Americans (AAs), the SNP8 G/G genotype was never observed in any of the control subjects in Sample 1 (n = 48), the newly-recruited unrelated controls in Sample 2 (n = 125), or the additional related unaffected pedigree subjects (n = 128; see Table 3) (i.e., in total there were 0/301 observations); this genotype was, however, observed in unrelated cases (e.g. 4/370, for AD). We cannot completely exclude the possibility that the difference between cases and controls is attributable to sampling bias and the findings are false positive, but we conjecture that it is more likely that this genotype is related to phenotype in AAs, although there is not enough information in this observation alone to support such a conclusion. Because this genotype is so rare (1%), some association methods with insufficient power might not detect a true genotype-phenotype association (in fact, evaluated by exact test as a case-control genotype frequency distribution comparison, this comparison shows p = 0.391 and 0.225 for AD vs. controls and DD vs. controls, respectively). Thus, in the present study, several association methods with differing power and that take different views of the data were applied and compared. The most powerful HWD test suggests that the *ADH4 *variant (rs2226896) might play an important role in risk for substance dependence (including AD and DD) in AAs, probably via a recessive genetic mechanism. The association between this variant and phenotype is population-specific, that is, it appears in AAs, but not in EAs. This association herein first discovered in AAs is a complementary finding to a previous set of genotype-phenotype relations we described for other markers at this locus in EAs [6,7]; based on this result, we can now provisionally conclude that *ADH4 *affects SD risk in both EAs and AAs, but different variants are important in the different populations. It would be of great interest to study this variant in other populations, e.g., Asians, to further characterize the population specificity we report here. This variant is independent of the other seven polymorphisms that were reported previously to have no association with substance dependence in AAs [6,7].

*ADH4 *gene variation is thought to influence the risk for AD by modulating ethanol metabolism. However, we find that it is associated with DD too. This is reasonable because DD has many features in common with AD which are reviewed above and because the development of AD and DD might have some similar pathophysiological mechanisms. ADH4 enzyme (*π *ADH) catalyzes synthesis from substrates (which include, e.g., norepinephrine aldehydes, including 3,4-dihydroxymandelaldehyde (DHMAL) and 4-hydroxy-3-methoxymandelaldehyde (HMMAL)) to create the intermediary glycols of norepinephrine metabolism, including 3,4-dihydroxyphenylglycol (DHPG) and 4-hydroxy-3-methoxyphenylglycol (HMPG), respectively. This catalysis is considerably more efficient via this isozyme than for any of the class I isozymes (α, β and γ ADHs); and class III ADH (χ ADH) does not have any detectable catalytic activity towards these substrates at all [37]. Increased *π *ADH activity – e.g., through genetic variation, such as, potentially, the SNP8 G/G genotype – could lead to increasing levels of DHPG and HMPG, and a very high turnover of norepinephrine aldehydes. To block the turnover of norepinephrine aldehydes, perhaps one might self-administer ethanol to compete DHPG and HMPG, because ethanol is an external competitor for internal DHPG and HMPG on *π *ADH [37]. This mechanism could lead to AD. Cocaine, which partially functions as a norepinephrine re-uptake inhibitor, can activate the noradrenergic system [38]. Plasma epinephrine and norepinephrine concentrations were significantly increased in response to cocaine injection [38]. Intravenous opioids stimulate norepinephrine and acetylcholine release in cerebrospinal fluid [39]. Therefore, self-administration of drugs (cocaine and opiates) could elevate norepinephrine aldehydes too, which may lead to DD.

A family-based association study is immune from population stratification effects. Thus, in the present study, subjects with different ethnicities, including EA, AA, Hispanic, and others, and the affected and unaffected parents, were combined in the analysis, to increase the statistical power. Allowing for the possible population-specificity of association, we also performed this analysis separately within EAs and AAs. However, the family-based association studies revealed no significant association between this *ADH4 *variation and substance dependence (both in Samples 3 and 4). This is likely due to the limited statistical power, given the small sample size (Sample 3: 115 affected offspring and 221 parents), in the context of the fact that the SNP8 variant has a rare minor allele (Sample 3: frequency ≤ 0.049 in AAs and ≤ 0.077 in EAs; Sample 4: frequency ≤ 0.020 in AAs and ≤ 0.053 in EAs;). Additionally, only heterozygous parents yield TDT information, which further limits the power for the family-based association studies.

In the present study, our case-control sample (820 cases; 483 controls) has approximately five times the power of the family Sample 3 [10]. However, neither allelewise nor genotypewise case-control comparisons showed any significant association between *ADH4 *variation and substance dependence. The case-control design is theoretically vulnerable to population stratification that could result in false negative findings. We therefore used the structured association (SA) method [15] to exclude population stratification and admixture effects on associations. The results did not change substantively after controlling for population stratification and admixture effects, i.e., both were negative. Similarly, despite taking into account the potential confounding effects of age and sex via regression analysis, no association between this variation and phenotypes was detected. Additionally, the low detected admixture degrees in EAs (1.6%) and AAs (3.9%) (which may have appeared especially low, particularly in the AAs, because of lack of inclusion of an ancestral African population) suggest that admixture effects should not have substantially affected the analysis in this study. It is possible that the negative findings from the case-control association might, like the TDT analysis and the FBAT analysis, result from insufficient power.

For this particular marker, the allele frequency of the rare allele is higher for EAs than for AAs (0.077 vs. 0.046, for the control subjects). EAs are therefore expected to have a substantially higher frequency of rare homozygotes than AAs – 0.006 vs. 0.002, i.e. about three times as many. Therefore, we specifically considered possible European admixture in the four homozygous AA patients. We found that the European ancestry proportions in these four AA subjects were less than 0.72%, indicating these observations of the rare homozygote are unlikely to be related to the genomewide European admixture in these AA subjects.

HWD at SNP loci in the case sample could be an indicator of gene-phenotype association [7,9,35-42]. Cases are ascertained due to their "affected" status, so disease susceptibility genotypes or alleles should be present at high rates in the case sample, which might violate HWE. Further, because cases are not randomly sampled from the general population where there is random mating and 2N alleles among N subjects are independent, HWE of disease-related marker loci in cases could be violated, and 2N alleles could become dependent. Only when the marker has no LD with the disease locus, i.e., the marker genotype frequency distributions are independent of the diagnosis, can the case group and the control group have the same genotype frequency distributions, with both in HWE. Therefore, the HWD of SNP8 in AD and DD among AAs in the present study suggests an association between SNP8 and both AD and DD. Usually, susceptibility loci are in HWD in cases, but in HWE in controls [7], as observed for SNP8. This is because a much greater sample size is needed to detect HWD in controls than in cases [9]. If the predisposing effect of the disease susceptibility allele is strong enough and the sample size for controls is large enough, this locus could also be in HWD in controls, but with an excess of the protective genotype, the opposite of the situation for cases [9]. SNP8 does not, apparently, have a strong enough effect on risk to distort HWE in controls; alternatively, the size of the control sample is not large enough to detect HWD in that sample.

Additionally, substance dependence significantly increases mortality [43-45], leading to age cohort-related dropout of the disease-associated genotypes or alleles from the population (i.e., natural selection). Selection by mortality may violate an assumption for HWE and cause altered distribution of genotype frequencies (i.e., HWD) [46]. This dropout makes the risk genotype or allele rarer, but the risk genotype or allele is still more common in cases than in controls, consistent with what we observed for SNP8, and providing additional evidence that SNP8 might be a disease-associated locus.

The magnitude of the HWD test statistic varies with the distance between the marker loci and the disease locus; that is, deviations from HWE are greatest at trait susceptibility loci, and can also be detected for benign polymorphisms that are in LD with the susceptibility locus [35,40]. In the present study, SNP8 is in HWD in cases, which suggests that the risk locus for substance dependence might be located in the SNP8-containing haplotype block or be SNP8 itself.

The association evident from the HWD test was not detected by case-control frequency comparison. This is because the HWD test as an association method is, sometimes, much more powerful than case-control comparison [7], for which, the present study provides an extreme example. One reason is that, from a statistical perspective, the HWD test in cases has one degree of freedom (*df *= 1), rather than *df *= 2 for case-control genotype frequency comparisons. Another reason for greater power of the HWD test in the present study is the age difference between cases and controls, from an epidemiological viewpoint, as discussed in our previous study [7]. In this study, the average age for controls was 29.7 years, about 10 years younger than that for cases, 39.6 years. Many healthy controls, although presently unaffected, have not completely passed through the age of risk to manifest AD or DD. The healthy controls have a probability (≈ lifetime prevalence of disease; less than the cumulative prevalence by the subject's age) to develop disease at some point in the future, and this probability increases with the residual prevalence, so that the case-control association design may be less powerful than a case-only study using the HWD test. That is, some associations that can be discovered by the use of a case-only study might not be detected using a case-control design. Meanwhile, because the dropout of disease-related genotypes or alleles increases with the age of cases (due to increasing mortality), an HWD test that reflects the dropout could be more sensitive to detect this disease-related locus, especially when cases are much older than controls.

The HWD signal of a marker locus decays more rapidly with distance from a causative locus than the LD signal [35]. The closer a marker is to the causative locus, the greater the excess of power for the HWD test over the LD test. Nielsen et al. [40] demonstrated that the HWD method was more powerful than the LD method under certain conditions (recessive and additive models), which was also supported by many other studies [7,9,35,47-49]. Kocsis et al. [50] demonstrated that even in the absence of significant differences in genotype frequency distribution between cases and controls, associations can be detected by HWD, as observed for SNP8 in present study. This is particularly true for a trait-associated marker that acts via a recessive mode of inheritance, because the effect of the recessive allele (i.e., the disease-risk allele) can be "masked" by the dominant allele (i.e., the non-disease-risk allele), which yields negative results in case-control frequency comparisons [under HWD, the two alleles are dependent and affect each other]. From the formula of goodness-of-fit χ^2 ^test for HWD, the χ^2 ^value (HWD statistic) is proportional to the sample size (N) and the squared difference between observed genotype frequency and the expected frequency (Δ^2^), and inversely proportional to the expected genotype frequencies. Thus, even when the Δ is small, one rare genotype frequency could generate a high HWD statistic. If the expected count of this rare genotype is less than 5, we use an exact test; the exact p value is usually consistent with that from the goodness-of-fit χ^2 ^test, as seen in additional file 1. This is why HWD test is especially sensitive to a marker with one rare genotype.

HWD might not be more powerful than LD method in detecting gene-disease association when a trait-associated marker acts via a multiplicative mode of inheritance, because HWD test would have very little power under this disease model [35,40,41,47-49,51]. However, SNP8 unlikely acts via a multiplicative model in the present study (p ≤ 0.007, see additional file 1). A new method, the weighted average (WA) statistic test, has been reported to be even more powerful than the HWD test to detect association between disease susceptibility and marker loci under many genetic inheritance models, including the recessive, additive and multiplicative models [49]. However, application of this method is beyond the scope of the present study.

The HWD test can not only detect gene-phenotype association, but can also reflect a genetic disease model, because the direction of HWD statistics (Δ, F and J) varies with the genetic model [9,35,36]. In the present study, the Δ, F and J for SNP8 are positive, suggesting that SNP8 appears to follow a recessive genetic disease model.

We also identified the genetic disease model for SNP8 with the best fit to the genotypic proportions observed in patients and controls using the Mathematica Notebooks written by Wittke-Thompson et al. [9]. Consistent with the above inference, the "best-fit" model for SNP8 is a recessive model. This model-fitting method can not only identify the genetic model, but can also tell us that other explanations for the observed HWD, including chance, genotyping error, and/or violations of the requisite assumptions of HWE, are less likely, if one "best-fit" model can be identified [9]. However, it should also be noted that just because an observed HWD is consistent with a "best-fit" genetic model does not completely guarantee that errors, missing data patterns, or violations of HWE assumptions do not generate or contribute to the observed HWD. Actually, HWD can be attributable to a combination of factors [9]. But the following analyses further support the interpretation that non-disease factors underlying HWD are less likely to be important in explaining our data. We note, though, that it might take a very large sample to fully support this conclusion.

Signal intensity, background noise, and clustering properties all play a role in the ability to assign genotypes correctly, and in determining the types of errors that occur [52]. Genotyping error is one of the greatest concerns for causing spurious HWD observation. First, DNA contamination can result in the lack of one homozygote in the PCR product, which leads to a deficiency of observed homozygotes [52]. This runs counter to our data and thus is not a possible explanation for the present study. Second, incomplete digestion of PCR product (relevant only when using the RFLP technique, not the TaqMan technique) or poor amplification of one of the alleles will lead to heterozygous genotypes being read as homozygous genotypes [53]. This kind of allele dropout can lead to an excess of apparent homozygous genotype observations, which does fit our data and thus needs to be considered. Also, when genotypes are read, heterozygote genotypes could, theoretically, be more ambiguous, and therefore more likely to be scored as "missing," than homozygote genotypes. To detect possible genotyping error, for family data, we assessed the data for Mendelian consistencies by the program PEDCHECK [54], with no non-Mendelian inconsistencies detected. For all subjects, including family and case-control subjects, we also replicated the genotypes (the most accurate way to estimate genotyping error rates), so that all genotypes were matched. Missing genotype data rate was not significantly different between cases and controls. Additionally, controls were tested for HWE and did not show the same direction of HWD statistics as cases. Together, the evidence suggests that genotyping error as an explanation for the observed HWD is improbable.

Violation of one of the other HWE assumptions (besides selection of alleles by disease) can also cause HWD. First, genetic drift can cause HWD. Genetic drift is the effect of finite population size [55], such that the smaller the population, the more noticeable the effects of drift. All populations are finite and all genetic variation is subject to genetic drift. In a finite population, allele frequencies fluctuate by chance randomly and the fluctuation leads to deviations from HWE (in this context, this is "sampling error") [56]. If a population is small enough, the effects of drift may overwhelm the other forces described below, even selection. Our AA case sample is large enough such that genetic drift at the disease susceptibility locus and the marker locus can reasonably be ignored. Additionally, in our AA control sample, which is smaller than that of cases, HWE was not violated. Further, our AA samples are representative of the general population [57], which supports the interpretation that HWD in AA cases is probably not due to a sample size issue; however, considering the small number of homozygote observations that are critical in driving the finding, we cannot exclude this possibility. Second, inbreeding can cause HWD. Inbreeding is a type of positive assortative mating which is non-random. Most populations are geographically divided, and mating is local, so inbreeding could be common, but to varying extents. During inbreeding, individuals are more likely to mate with relatives than with non-related individuals. One common consequence of inbreeding is that the number of heterozygotes decreases and the number of homozygotes increases [58], which leads to HWD. Another common consequence of inbreeding is that the expression of deleterious recessive alleles in the population increases, which reduces average fitness and increases mortality ("inbreeding depression") [59], which, as described above, can also contribute to HWD. However, our case and control samples are ascertained as unrelated, and we have no evidence for the existence of overlapping generations, making inbreeding unlikely in the present study. Third, gene flow may result in HWD. Gene flow is the result of migration. Immigrants carrying new alleles into the population may change the genotype frequency distribution of that population with resulting HWD in that generation. Contrary to selection and genetic drift, gene flow eventually homogenizes allele frequencies among populations. Although gene flow occurs in most populations, its contribution to major shifts in allele frequencies is usually negligible. The AA population has been in the US for an average of about five generations and we do not have evidence of major immigration for the current AA generation, so gene flow resulting in HWD in our AA sample is unlikely. Fourth, mutation may increase the genetic variability due to genetic drift and might cause HWD. But because change in allele frequencies induced by mutation is so small from one generation to the next, we can safely ignore mutation as a factor in HWD. Unless mutation rates are abnormally high, for which we have no evidence in the present data, the change in allele frequencies is believed to be virtually nil. In conclusion, violation of one of the above HWE assumptions causing HWD is unlikely. However, the caution that these results are driven primarily by a small group of subjects, and that our conclusions would be different if just a few of them were omitted or somehow changed diagnosis, bears repeating; this reliance on a small number of subjects requires us to be very tentative in our conclusions.

In addition to the factors that have been discussed, other factors can also cause HWD. For example, population stratification and admixture can cause HWD, as demonstrated by Luo et al. [6]. However, we have demonstrated that the admixture degrees in our EA and AA samples are relatively low, suggesting that this factor can be ignored as the sources of HWD in our case samples. In addition, the cases and the controls are drawn from the same populations, but the controls are in HWE for SNP8, reducing the possibility that HWD in cases results from an effect of admixture. Nonrandom patterns of missing data may also generate a relatively consistent pattern of HWD (e.g., disproportionate missing data in heterozygotes may lead to a consistent pattern of HWD, with an excess of homozygotes, as observed for SNP8). This possibility is common and inevitable and thus should not be ignored. However, our cases and controls were genotyped using same genotyping systems, reducing the possibility that non-random patterns of missing data cause HWD only in cases but not in controls. Further, if the cases or controls are old enough, the marker can be in HWD because a specific mortality-related allele drops out due to death associated with advancing age [51]. However, the ages of our subjects range from 17 to 78 years, making this explanation unlikely. An unrecognized polymorphism in primer sequences used in PCR may also lead to HWD, with an excess of homozygotes, as observed for SNP8, particularly when the primer polymorphism is in LD with the tested marker [9]. Finally, genomic duplications or deletions (a copy deletion could lead to hemizygosity) can also lead to HWD [9]. We believe that these explanations are not appropriate to explain our data in the present study, but these factors can be excluded only through extensive sequence analysis.

In conclusion, the presence of HWD for SNP8 suggests that this polymorphism might be a risk locus for substance dependence in AAs, although our direct evidence for this conclusion is weak and the false positivity cannot completely excluded. SNP8 is located at the putative 5' regulatory region of *ADH4*. It might indirectly modulate risk for disease via LD with an unknown nearby functional variant, e.g., in ABI and HapMap database, it is 2.5 kb far from and in LD with rs7434491 which could significantly alter the secondary structure of *ADH4 *mRNA (IDT SciTools: ); it might also alter the transcription initiation site or the capacity of transcription factors to bind to the DNA sequence, and consequently, directly affect transcription levels; it might result in mRNA instability, altered translational efficiency, or even different protein expression levels in different tissues. Considering our sample size limitations, we believe that replication of these results is critical. Nevertheless, given our findings, we believe that it would be productive to study the effect of this variation directly on protein expression, in order to provide convergent validation of the findings reported here and to elucidate the specific mechanism underlying the association of SNP8 at *ADH4 *to both AD and DD.

## Limitations

The limitations of the present study have been discussed above. In summary, in the present study, because the risk genotype is rare, some analytic approaches including the goodness-of-fit test might not have sufficient power in detecting associations; how conservative the correction for multiple testing should be remains disputed; and the function of the risk marker remains unclear. Allowing for these limitations, the possibility that the findings are false positive cannot be completely excluded. Future replication studies with stricter design and improved sampling methods, and increased study power are warranted.

## Competing interests

The authors declare that they have no competing interests.

## Authors' contributions

XL and LZ designed and coordinated the study. XL and LZ drafted the manuscript. LZ and SW participated in data management and statistical analysis. RFA participated in sample collection and provided critical comments. JG and HRK participated in the supervision of the project. All authors read and approved the final manuscript.

## Supplementary Material

Additional file 1 Genotype and allele frequencies of SNP8 and p-values for genetic model fitting. Click here for file

Additional file 2 Transmitted and non-transmitted allele frequencies in small nuclear families. Click here for file
