# Supplementary material for: Recessive genetic mode of an ADH4 variant in substance dependence in African-Americans: A model of utility of the HWD test
Source: Behav Brain Funct. 2008 Sep 18;4:42. doi: 10.1186/1744-9081-4-42 (PMC2563013; doi:10.1186/1744-9081-4-42)
Supplement: Additional file 1 — Genotype and allele frequencies of SNP8 and p-values for genetic model fitting. [file 1744-9081-4-42-S1.doc]

Genotype and allele frequencies of SNP8 and p-values for genetic model fitting (Samples 1 and 2)

|  | European-Americans . | | | | | | | | | | | | African-Americans . | | | | | | | | | | | |
| --- | --- | --- | --- | --- | --- | --- | --- | --- | --- | --- | --- | --- | --- | --- | --- | --- | --- | --- | --- | --- | --- | --- | --- | --- |
|  | Alcohol dependence . | | | | Drug dependence . | | | | Controls . | | | | Alcohol dependence^1^ . | | | | Drug dependence^2^ . | | | | Controls . | | | |
|  | *N_o_* | *f_o_* | *N_e_* | *f_e_* | *N_o_* | *f_o_* | *N_e_* | *f_e_* | *N_o_* | *f_o_* | *N_e_* | *f_e_* | *N_o_* | *f_o_* | *N_e_* | *f_e_* | *N_o_* | *f_o_* | *N_e_* | *f_e_* | *N_o_* | *f_o_* | *N_e_* | *f_e_* |
| A/A | 280 | 0.859 | 281 | 0.861 | 178 | 0.873 | 177 | 0.867 | 264 | 0.852 | 264 | 0.851 | 338 | 0.914 | 335 | 0.905 | 201 | 0.931 | 199 | 0.923 | 157 | 0.908 | 157 | 0.910 |
| A/G | 45 | 0.138 | 44 | 0.134 | 24 | 0.118 | 26 | 0.128 | 44 | 0.142 | 44 | 0.143 | 28 | 0.076 | 34 | 0.093 | 13 | 0.060 | 16 | 0.076 | 16 | 0.092 | 15 | 0.088 |
| G/G | 1 | 0.003 | 2 | 0.005 | 2 | 0.010 | 1 | 0.005 | 2 | 0.006 | 2 | 0.006 | 4 | 0.011 | 1 | 0.002 | 2 | 0.009 | 0 | 0.002 | 0 | 0.000 | 0 | 0.002 |
| A | 605 | 0.928 |  |  | 380 | 0.931 |  |  | 572 | 0.923 |  |  | 704 | 0.951 |  |  | 415 | 0.961 |  |  | 330 | 0.954 |  |  |
| G | 47 | 0.072 |  |  | 28 | 0.069 |  |  | 48 | 0.077 |  |  | 36 | 0.049 |  |  | 17 | 0.039 |  |  | 16 | 0.046 |  |  |
| ^3^Recessive |  |  |  |  |  |  |  |  |  |  |  |  | 0.733 | | | | 0.450 | | | |  |  |  |  |
| ^3^Dominant |  |  |  |  |  |  |  |  |  |  |  |  | 0.004 | | | | 0.026 | | | |  |  |  |  |
| ^3^Additive |  |  |  |  |  |  |  |  |  |  |  |  | 0.117 | | | | 0.174 | | | |  |  |  |  |
| ^3^General |  |  |  |  |  |  |  |  |  |  |  |  | 0.733 | | | | 0.450 | | | |  |  |  |  |
| ^3^Multiplicative |  |  |  |  |  |  |  |  |  |  |  |  | 0.001 | | | | 0.007 | | | |  |  |  |  |

^1^In African-Americans with alcohol dependence, p-value for HWE test is 0.0071 (exact test); ^2^in African-Americans with drug dependence, p-value for HWE test is 0.0341 (exact test). ^3^These are the genetic disease models tested by WT goodness-of-fit chi-square test: only the models in the subgroups where the marker is in HWD were tested and the corresponding empirical p-values were listed. There is no significant difference in allele or genotype frequency distributions between cases and controls in either EAs or AAs, either before or after controlling for admixture effects and confounding effects of age and sex (all p>0.05). *N_o_*, *f_o_*, *N_e_*, *f_e_*, the observed (o) and expected (e) individual numbers (for genotypes) or chromosome numbers (for alleles), and their frequencies (*f*), respectively. N/A, not applicable.
