# Supplementary material for: Recessive genetic mode of an ADH4 variant in substance dependence in African-Americans: A model of utility of the HWD test
Source: Behav Brain Funct. 2008 Sep 18;4:42. doi: 10.1186/1744-9081-4-42 (PMC2563013; doi:10.1186/1744-9081-4-42)
Supplement: Additional file 2 — Transmitted and non-transmitted allele frequencies in small nuclear families. [file 1744-9081-4-42-S2.doc]

Transmitted and non-transmitted allele frequencies in small nuclear families (Sample 3)

|  | Whole sample . | | | | | | | | European-Americans . | | | | | | | | African-Americans . | | | | | | | |
| --- | --- | --- | --- | --- | --- | --- | --- | --- | --- | --- | --- | --- | --- | --- | --- | --- | --- | --- | --- | --- | --- | --- | --- | --- |
|  | Alcohol dependence | | | | Drug dependence | | | | Alcohol dependence | | | | Drug dependence | | | | Alcohol dependence | | | | Drug dependence | | | |
|  | T . | | non-T . | | T . | | non-T . | | T . | | non-T . | | T . | | non-T . | | T . | | non-T . | | T . | | non-T . | |
|  | *N* | *f* | *N* | *f* | *N* | *f* | *N* | *f* | *N* | *f* | *N* | *f* | *N* | *f* | *N* | *f* | *N* | *f* | *N* | *f* | *N* | *f* | *N* | *f* |
| A | 126 | 0.913 | 129 | 0.935 | 133 | 0.923 | 134 | 0.931 | 104 | 0.912 | 109 | 0.956 | 104 | 0.929 | 107 | 0.955 | 13 | 0.929 | 11 | 0.786 | 17 | 0.944 | 14 | 0.778 |
| G | 12 | 0.087 | 9 | 0.065 | 11 | 0.076 | 10 | 0.069 | 10 | 0.088 | 5 | 0.044 | 8 | 0.071 | 5 | 0.045 | 1 | 0.071 | 3 | 0.214 | 1 | 0.056 | 4 | 0.222 |

Whole sample refers to the combination of European-Americans, African-Americans, Hispanics and other populations. T, transmitted allele; non-T, non-transmitted allele. *N, f,* chromosome numbers and their frequencies.
